# Sexual morphs of *Pterocomma tremulae* Börner, 1940 (Aphididae, Aphidinae) with description of male reproductive system

**DOI:** 10.3897/zookeys.686.14493

**Published:** 2017-07-25

**Authors:** Agnieszka Nowińska, Ewa Mróz, Łukasz Depa

**Affiliations:** 1 Zoology Department, Faculty of Biology and Environmental Protection, University of Silesia, Bankowa 9, PL 40–007 Katowice, Poland

**Keywords:** Aphid, phylogeny, *Populus*, reproduction, taxonomy

## Abstract

Paper presents the first description of the so far unknown sexual generation of *Pterocomma tremulae* (Aphididae, Aphidinae): oviparous female and alate male. It also provides detailed description of the male reproductive system. Discussion focuses on comparative analysis of male reproductive system with other aphid groups and possible importance of its structure in resolving phylogenetic interrelationships within the genus *Pterocomma*. A key is provided to the known males of European *Pterocomma* species.

## Introduction

Pterocommatinae have long been a somewhat problematic group within Aphididae, having been placed in most systems in close relationship with Aphidinae. The detailed history of classification has been presented in revision of Palaearctic species of Pterocommatinae made by Wojciechowski (2003). This taxon bears a set of distinct morphological traits, giving base to distinguish it from the subfamily Aphidinae. However, recent studies with application of molecular markers, both mitochondrial and nuclear, indicated the strong relationship of what was so far regarded as a subfamily Pterocommatinae, with aphid genera belonging to the “Liosomaphidine” branch of Aphidinae, within the tribe Macrosiphini (Tang et al. 2015) or close to Macrosiphini (Papasotiropoulos et al. 2013). The current concept of systematic position of “Pterocommatines” *sensu stricto*, is that it is part of tribe Macrosiphini (Blackman and Eastop 2017).

However, within the genus *Pterocomma* itself, there are also some problems concerning the very variable morphology of a few species, making it difficult to distinguish closely related species e. g. *Pterocomma pilosum* Buckton, 1879; *P. konoi* Takahashi, 1939; *P. dubium* Börner, 1950; *P. ringdahli* Wahlgren, 1940; or various subspecies, of uncertain taxonomical status e.g. *P. pilosum sarmaticum* Szelegiewicz, 1967 (Wojciechowski 2003, Mróz and Depa 2014, Blackman and Eastop 2017). Resolution of these doubts definitely requires detailed morphological and molecular studies, but this is possible only after proper recognition of crucial morphs of each form and species, especially the sexual generation (Wieczorek et al. 2011, Wieczorek et al. 2013, Kanturski et al. 2017). Application of anatomical studies of the reproductive system, especially the male reproductive system may also shed new light on species identity and systematic relationship (Wieczorek 2006).

*Pterocomma tremulae*, Börner 1940 is relatively well defined in terms of its morphological characteristics. There are some accounts of its intraspecific variation, but the species as whole may be well recognized as the only *Populus*-feeding species bearing few to many secondary rhinaria on antennal segment III, a trait occurring also only in *Pterocomma kozhuchovae*, most probably an eastern Palearctic vicariant species. Here we present the first description of the sexual morphs and also provide the first description of the male reproductive system of this species.

## Material and methods

Collection data of specimens applied for taxonomical description:

17.10.2014, Katowice; *Populus tremula*; leg. A. Nowińska, det. Ł. Depa, 2 oviparous females.

17.10.2014, Katowice; *Populus tremula*; leg. A. Nowińska, det. Ł. Depa, 2 alate males.

Also 29 specimens of alate males were collected from the same collection site to anatomical studies. The provisional key to males of European representatives of the genus Pterocomma was prepared on the basis of morphological descriptions and measurements given by Wojciechowski (2003).

### Anatomical studies

**Histological preparations**

In order to analyse the anatomical structure of the male reproductive system of *P. tremulae*, the paraffin method was applied. The material was collected into Eppendorf microtubes containing 70% ethanol for keeping and preserving collected specimens. Next, insects were dehydrated in increasing concentration of ethanol (90%, 96%, 100%). In order to ensure transparency, specimens were kept in methyl benzoate for one night. Then, material was consecutively transferred to benzene, benzene with paraffin (in proportions 2:3 and 1:2), paraffin I (melting point: 56°C) and finally to paraffin II (melting point: 60°C), where it was kept for the night. After this process, material was immersed in paraffin II. The bars obtained were sectioned into 5 µm strips, which were stuck on slides in a 0.5% gelatine solution at temperature 50–52°C. Then, the slides were dried in 37°C.

Slides were next deparaffined in xylene and treated with a series of ethanol solutions (100–60%). They were rinsed in distilled water, stained with Ehrlich’s acid hematoxylin for about 20 minutes, rinsed again and differentiated with xylidine ponceau. After this process, preparations were treated with series of ethanol solutions (60–100%), rinsed twice in xylene and embedded in Canadian balm or DPX.

This process was applied to 17 individuals of *P. tremulae*. The following histological preparations were prepared: cross-sections from 9 individuals and longitudinal sections from 8 individuals. In total 171 microscopic slides were made, including 84 preparation of cross-section and 87 of longitudinal section.

**Mounting of the whole tract**

The specimens of *P. tremulae* were put into a droplet of 30% ethanol, on the microscopic glass, and with the mounting needles the whole reproductive system was extracted from the body. The extracted organs were preserved in glycerol and mounted, to make all the anatomical structures visible with the stereomicroscope. A total number of 12 preparations were made using this technique.

The documentation was prepared using Nikon Eclipse E6000, with measurements made by Lucia net program. The pictures of translucent specimens were taken with a stereomicroscope equipped with monochromatic camera Axio Cam programmed with Axio Vision.

## Results

### Description of sexual morphs of P. tremulae

Taxon classificationAnimaliaHemipteraAphididae

, Börner, 1940

#### Oviparous female.

**Figure 1.**
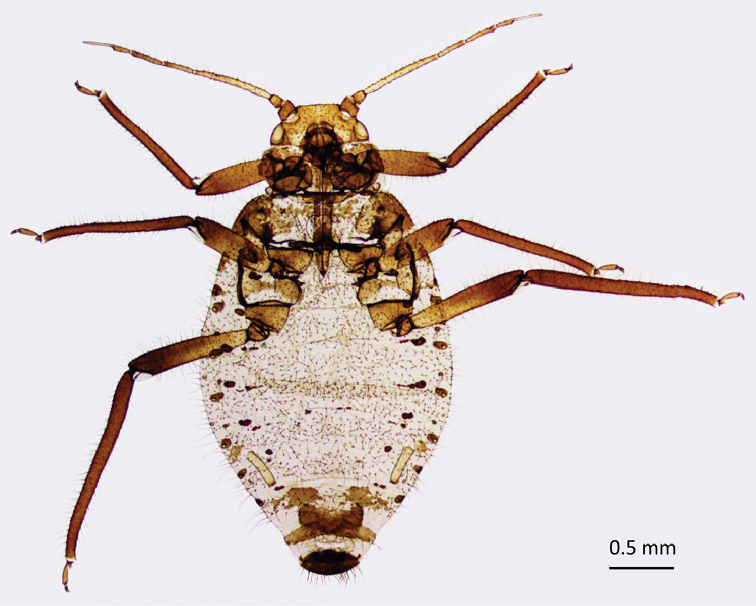
Oviparous female of *P. tremulae*.

**Figure 2.**
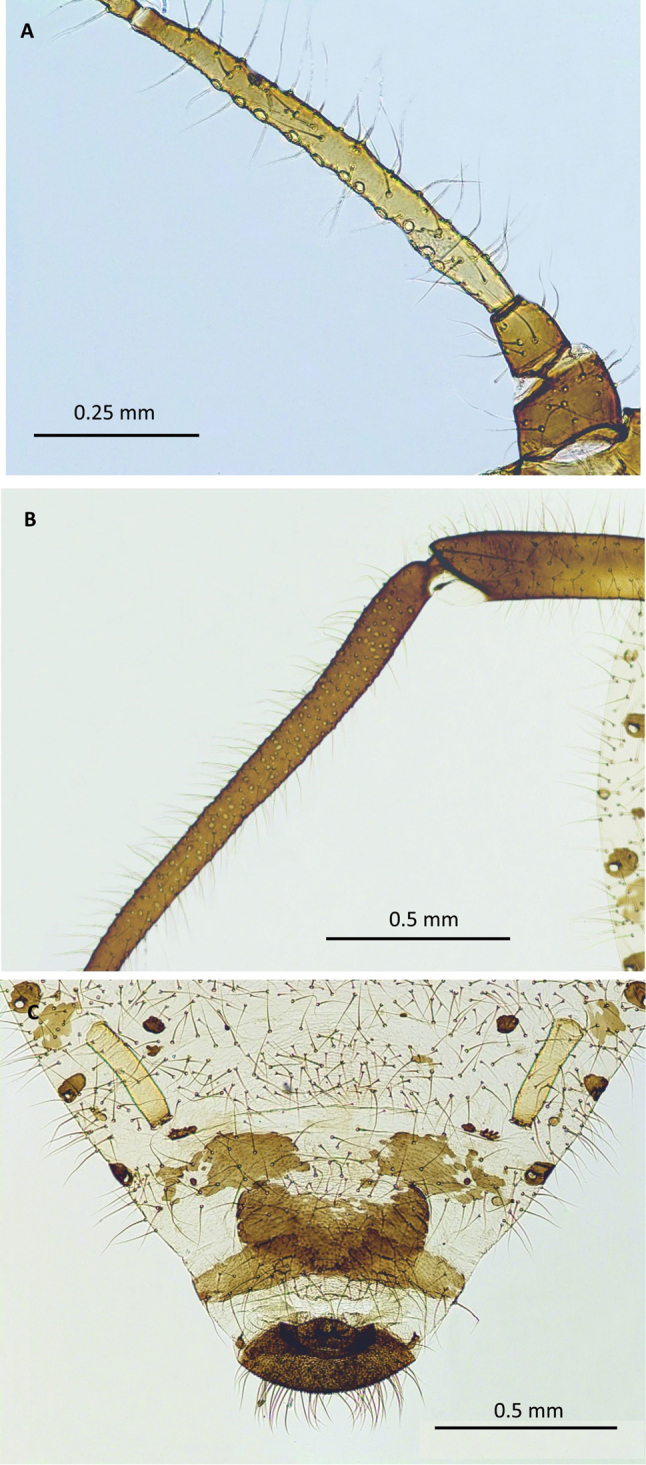
Morphological features of the oviparous female: **A** antennal segment III with secondary rhinaria **B** hind tibia with numerous scent plaques **C** posterior part of the abdomen, with siphunculi, cauda and genital plate.

Body oval, 3.66–4.03 mm long (Fig. 1), grey to brightly brown, significantly convex. Head and pronotum dark and sclerotised; mesonotum with marginal sclerites, and spinal sclerites broken into smaller scleroites in the middle; metanotum with weak marginal sclerites only. Mesothoracic furca separated, but with band of dark sclerotisation between them, giving the impression of being joined. Antenna 6-segmented, brownish, with base of antennal segment III paler (Fig. 2A), 1.72–1.92 mm long, 0.47–0.48 of body length. Antennal segment III 0.64–0.71, IV 0.26–0.29, V 0.26–0.31, VI 0.37–0.41 mm long; length of antennal segment III: 2.39–2.50 of the length of antennal segment IV, 2.29–2.44 of antennal segment V; processus terminalis 0.23–0.26 mm long, 1.60–1.90 of VIa. Antenna covered with long, erect hairs, 0.11–0.13 mm long. Number of secondary rhinaria on III antennal segment: 13–20 (Fig. 2), on IV antennal segment 0. Base of antennal segment VI with 6–8 erect hairs. Rostrum 0.90–1.10 mm long, 0.25–0.27 of body length, with ultimate rostral segment 0.22–0.23 mm long, 1.27–1.30 of second segment of hind tarsus, 0.33–0.34 of III antennal segment, 0.72–0.77 of siphunculus, with 12–13 accessory hairs (Fig. 1). Legs dark, with brighter bases of femora and coxae, second segment of hind tarsus 0.17–0.18 mm long; legs covered with erect hairs, their length on tibia 0.10–0.12 mm. Hind tibia swollen with ca. 150–200 scent plaques (Fig. 2B).

Abdomen membranous, covered with dense pubescence. Length of hairs on abdominal tergite V 0.17–0.19 mm. Spinal sclerites only on VI and VII segment: on VI weakly developed, broken into minute scleroites. Intersegmental sclerotic insertions dark. Marginal slerites present on abdominal tergites I and V, sometimes also on VI but broken into smaller scleroites. Marginal tubercles present always on abdominal tergites I-IV, sometimes also on V-VII, their diameter at most as the diameter of spiracles, always dark pigmented. Number of hairs on abdominal tergite VIII ca. 50, their length 0.16–0.17 mm. Cauda rounded, dark pigmented, covered with many hairs (Fig. 2C). Genital plate semicircular, dark pigmented, 0.47–0.50 mm wide, covered with many hairs. Siphunculi pale, cylindrical, but may be slightly swollen in the distal half (Fig. 2C), 0.28–0.32 mm long, slightly darker at the apex, with very delicate flange, 0.44–0.45 of the length of antennal segment III.

#### Alate male.

**Figure 3.**
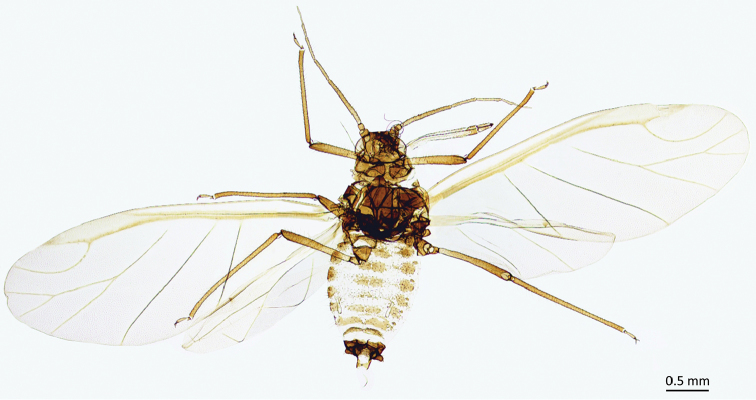
Alate male of *P. tremulae*.

**Figure 4.**
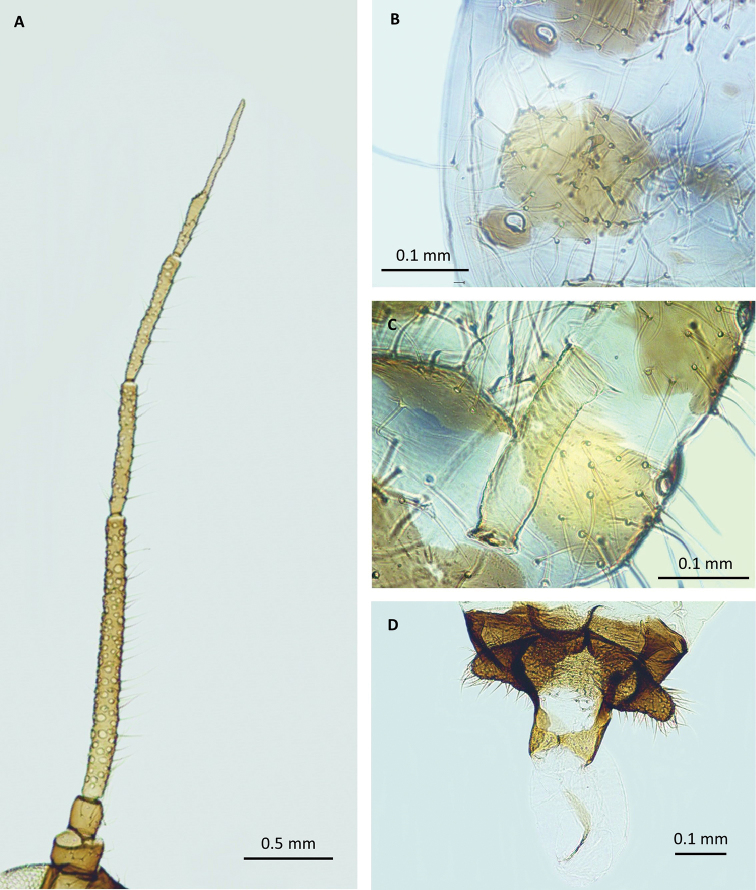
Morphological features of the male: **A** antenna with multiple secondary rhinaria **B** marginal plates and stigmal pores **C** siphunculus **D** genital apparatus with parameres and membranous aedeagus.

Body dark, brownish, 2.87–3.03 mm long (Fig. 3); head sclerotized, with ocelli well developed and big, multifaceted eyes with triommatidium. Antennae 6 segmented (Fig. 4A), 1.74–1.92 mm long, 0.61–0.65 of body length, brownish, only slightly paler than legs. Antennal segment III 0.63–0.71, IV 0.29–0.33, V 0.29–0.33, VI 0.38–0.44 mm long; length of antennal segment III: 1.62–1.63 of the length of antennal segment IV, 2.12–2.13 of antennal segment V; processus terminalis 0.22–0.27 mm long, 1.43–1.74 of VIa. Antenna covered with long, erect hairs, 0.09–0.11 mm long. Number of secondary rhinaria on antennal segment III: 73–105 (Fig. 4A), on antennal segment IV: 27–43, V: 20–26, VI: 0–2. Base of antennal segment VI with 8–10 erect hairs. Rostrum 1.15–1.22 mm long, 0.38–0.42 of body length, with ultimate rostral segment 0.19–0.21 mm long, 1.17–1.25 of second segment of hind tarsus, 0.29–0.31 of III antennal segment, 0.94–1.00 of siphunculus, with 11–13 accessory hairs. Thorax heavily sclerotized, winged. Forewings with veins weakly sclerotized, medial vein branched twice. Legs uniformly brownish, only bases of femora slightly paler, covered with erect hairs 0.12–0.13 mm long. Second segment of hind tarsus 0.15–0.17 mm long.

Abdomen membranous, covered with hairs 0.010–0.015 mm long, with spino-pleural sclerites on abdominal segments I-VII often divided in the middle, and on segment V broken into smaller slceroits. Marginal sclerites present on all abdominal segments, with spiracles in the anterior margins of the sclerites and with conspicuous marginal tubercles (Fig. 4B), dark pigmented, on segments I-IV and VI-VII, occasionally also on V. Siphunculi pale, 0.19–0.22 mm long, 0.30–0.32 of antennal segment III, tapering, with weakly swollen apical part and small flange (Fig. 4C). Cauda small, pigmented, semicircular, sparsely covered with hairs. Genital apparatus sclerotized, parmeres conspicuous, dark, densely covered by relatively short hairs; base of the membranous aedeagus less sclerotized, sparsely covered by very short hairs (Fig. 4D).

### Description of the male reproductive system of *Pterocomma tremulae*

**Figure 5.**
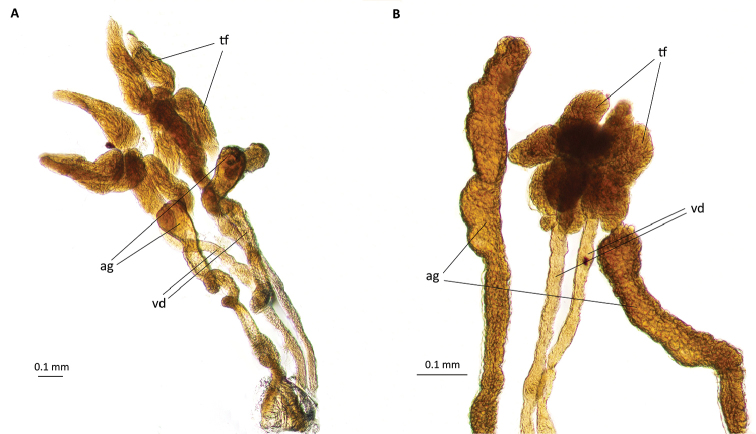
Morphology of the male reproductive system of *P. tremulae*: **A** young specimen **B** mature specimen; ag – accessory gland, tf – testicular follicles, vd – vas deferens.

**Figure 6.**
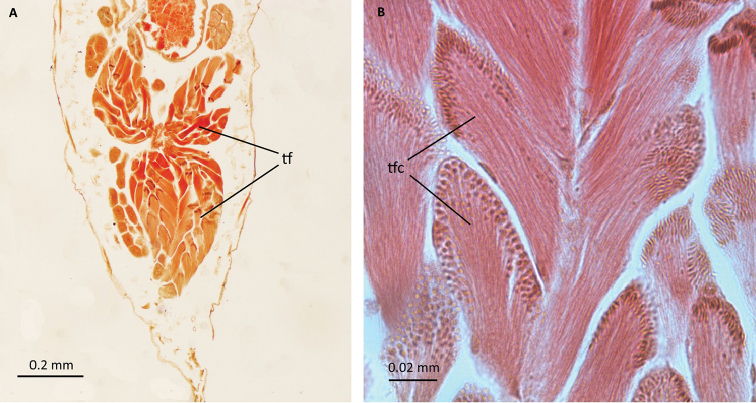
Male reproductive system of *P. tremulae*: **A, B** cross sections through testes; tf – testicular follicles, tfc – testicular follicle cysts.

The male reproductive system of *Pterocomma tremulae* extends parallel to the longitudinal axis of the body. It consists of two testes, located in the area of abdominal segments II and III, and a pair of accessory glands (Figs 5A, B, 6A). The total length of the system is 1.12–1.36 mm. One testis is 0.34–0.82 mm long and 0.26–0.44 mm wide. Each testis consists of four elongated follicles with rounded tips, having a diameter of 0.03–0.2 mm each. Each follicle contains cysts with differentiating male germ cells (Fig. 6A, B). Follicles connect to each other only at the base. They are located at the top of vas deferens, which they are connected to by short efferent ducts. Follicles of both testes can be also connected to each other. The length of one vas deferens is 0.68–1.12 mm, while its diameter 0.03–0.07 mm. Over the entire length it is more or less the same width.

**Figure 7.**
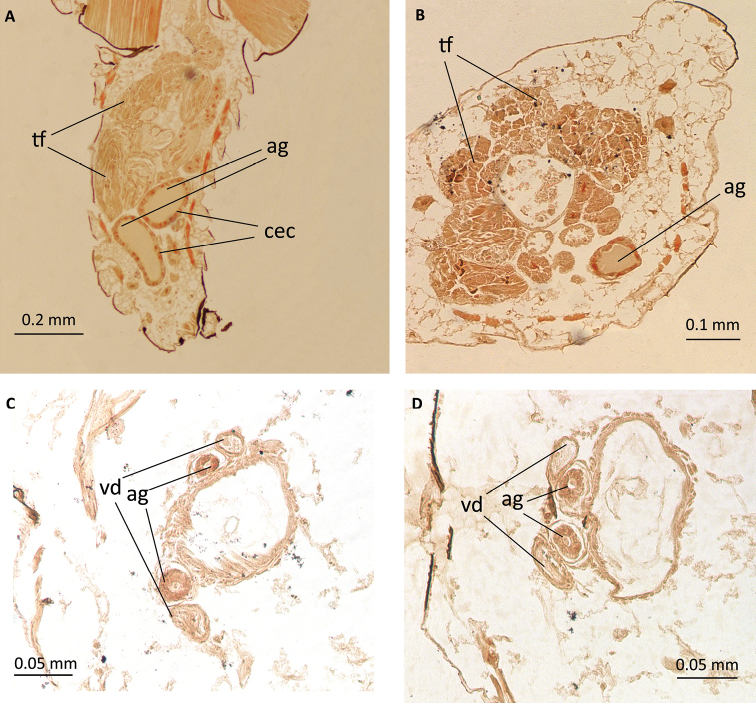
Male reproductive system of *P. tremulae*: **A** longitudinal section through testicles **B, C, D** cross sections through testicles; ag – accessory gland, cec – cuboidal epithelial cells, tf – testicular follicles, vd – vas deferens.

Accessory glands, of ectodermal origin, are 0.6–1.06 mm long. In examined species they are club-shaped or elongated. At the widest point their diameter is 0.05–0.12 mm, while at the narrowest point the diameter is 0.04–0.07 mm. Glandular wall is built of cuboidal epithelium (Fig. 7A, B). The height and width of the cells is 0.01 mm.

Depending on the age of the individual, testes and glands change their size. In younger individuals we observed testes several times larger than glands(Fig. 5A), while in older ones, testes are reduced and glands are enlarged (Fig. 5B). The vas deferens and the terminal sections of accessory glands extend next to each other independently and at the end they open to the ejaculatory duct (Fig. 7C, D).

### Key to known males of European species of the genus *Pterocomma*:

**Table:** 

1	Male apterous	***P. jacksoni***
–	Male alate	**2**
2	Siphunculi strongly swollen medially	***P. salicis***
–	Siphunculi cylindrical, at most slightly swollen distally	**3**
3	Antenna 0.6 of body length or longer	**4**
–	Antenna less than 0.6 of body length	**6**
4	Less than 50 secondary rhinaria on antennal segment III, on *Salix* spp.	***P. konoi***
–	More than 70 secondary rhinaria on antennal segment III, on *Populus* spp.	**5**
5	Less than 20 secondary rhinaria on antennal segment V	***P. dubium***
–	20 or more secondary rhinaria on antennal segment V	***P. tremulae***
6	Up to 15 secondary rhinaria on antennal segment IV	***P. rufipes***
–	18 or more secondary rhinaria on antennal segment IV	**7**
7	80 or less secondary rhinaria on antennal segment III	***P. pilosum***
–	80 or more secondary rhinaria on abdominal segment III	***P. populeum***

## Discussion

The male reproductive system of around 80 aphid species has been studied so far (Wieczorek et al. 2011). Compared to other insects, the characteristic features of aphids are the lack of seminal vesicles and the disordered arrangement of spermatids in the testicular follicles. The accessory glands are usually even in number, like in *Pterocomma tremulae* (Fig. 5A, B). They enter the ejaculatory duct independently of the vasa deferentia (Fig. 7B, C, D). The exception is in the Lachninae subfamily, which does not have accessory glands (Wieczorek and Świątek 2008, Wojciechowski 1977). However, in all the other studied species, accessory glands were present, entering the ejaculatory duct independently of vasa deferentia (Wieczorek 2006, Wieczorek and Świątek 2007 and 2008, Vitale et al. 2009). The ejaculatory duct is relatively short in most species, something that was also observed in *Pterocomma tremulae*. Its extremity is connected to the copulatory organ (Wieczorek and Świątek 2008).

In Aphidoidea, the number of testicular follicles varies between 2 and 7 (Szelegiewicz and Wojciechowski 1985). The number of follicles is not a feature characteristic of any one family, but has evolutionary significance. It allows the determination of the phylogenetic position of the aphid species. The smaller the number of follicles it has, the phylogenetically younger is the species (Wojciechowski 1977). The reduction of the number of testicular follicles is also related to the transfer of spermatogenesis to early larval stages, or in some species, to the termination of that process already in embryonic development (Blackman 1987). The initial number of testicular follicles is 7, as it is the number of pregenital segments. The number of follicles is reduced during oligomerization processes that proceed independently in each family (Mróz 2007). This number can also increase during the polymerization process. In such cases, the number of testicular follicles is 8 (Mróz and Wojciechowski 2011).

There are four testicular follicles in each testicle in *Pterocomma tremulae* (Figs 5, 6A). The male reproductive system of two other species of the genus *Pterocomma* has also been studied. It has been shown that *Pterocomma populeum* has 6 testicular follicles (Wieczorek and Mróz 2006), whereas *Pterocomma salicis* (Linnaeus, 1758) has 5 (Szelegiewcz and Wojciechowski 1985). That could indicate that *P. tremulae* belongs to the youngest evolutionary line within the genus *Pterocomma*. However, due to lack of data regarding the male reproductive system of many species from this genus, it is difficult to make a more precise analysis of this theory. It is not known whether there are two developmental lines involved in the adaptation to two host genera: *Populus* and *Salix*. *P. tremulae* is associated with *Populus*, as well as *P. populeum*, which has up to 6 testicular follicles. However *P. salicis*, which has 5 follicles, feeds on *Salix*. This incongruence requires explanation by studies on the male reproductive system of more species, including *Plocamaphis* spp. as an outgroup, supported by results of molecular studies.

## Supplementary Material

XML Treatment for P. tremulae

## References

[B1] Blackman RL (1987) Reproduction, cytogenetics and development. In: Minks AK Harrewijn P (Eds) Aphids: Their Biology, Natural Enemies and Control. Elsevier, Amsterdam 2: 163–195.

[B2] Blackman RL Eastop VF (2017) Aphids on the World’s plants. http://www.aphidsonworldsplants.info/index.htm [access: 19.05.2017]

[B3] Kanturski M Akbar ShA Favret C (2017) The Bhutan Pine Aphid Pseudessigella brachychaeta Hille Ris Lambers (Hemiptera: Aphididae: Lachninae) From India Reveals the Hitherto Unknown Oviparous Female and Dwarfish Male. Zoological Studies 56: 12 http://doi.org/10.6620/ZS.2017.56-12 10.6620/ZS.2017.56-12 PMC6517770 31966211

[B4] Mróz E (2007) Anatomical and molecular studies of Stenodema Laporte genus (Heteroptera: Miridae). Genus International Journal of Invertebrate Taxonomy 14: 77–81.

[B5] Mróz E Depa Ł (2014) Description of Fundatrices of Pterocomma tremulae Börner, 1940 and Pterocomma Pilosum buckton, 1879 (Sternorrhyncha: Aphididae). Annales Zoologici 64(1): 51–55. https://doi.org/10.3161/000345414X680564

[B6] Mróz E Wojciechowski W (2011) The systematic position the tribe Stenodemini (Heteroptera: Cimicomorpha: Miridae: Mirinae) in the light of the male internal reproductive system. Journal of Natural History 45(25–26): 1563–1588. http://dx.doi.org/10.1080/00222933.2011.559595

[B7] Papasotiropoulos V Tsiamis G Papaioannou Ch Ioannidis P Klossa-Kilia E Papapanagiotou A Bourtzis K Kilias G (2013) A molecular phylogenetic study of aphids (Hemiptera: Aphididae) based on mitochondrial DNA sequence analysis. Journal of Biological Research-Thessaloniki 20(1).

[B8] Szelegiewicz H Wojciechowski W (1985) The male internal reproductive system of aphids. In: Szelegiewicz (Edt) Evolution and biosystematics of aphids. Proceedings of the International Aphidological Symposium at Jabłonna, 5–11 April, 1981. Zakład Narodowy im. Ossolińskich, Wrocław, 239–244.

[B9] Tang X-J Jiang L-Y Chen J Qiao G-X (2015) DNA barcoding of Pterocommatinae (Hemiptera: Aphidoidea: Aphididae) based on three molecular markers. Acta entomologica sinica 58: 1262–1272.

[B10] Vitale DGM Brundo MV Sottile L Viscuso R Babbagallo S (2009) Morphological and ultrastructural investigations of the male reproductive system in aphids: observations on Tuberculatus (Tuberculoides) eggleri Börner (Hemiptera Aphidoidea). Redia 92: 195–197.

[B11] Wieczorek K (2006) Anatomical investigations of the male reproductive system of five species of Calaphidinae (Hemiptera, Aphidoidea). Insect Systematics & Evolution 37(4): 457–465. https://doi.org/10.1163/187631206788831434

[B12] Wieczorek K Kanturski M Junkiert Ł (2013) Shenahweum minutum (Hemiptera, Aphidoidea: Drepanosiphinae) – Taxonomic position and description of sexuales. Zootaxa 3731(3): 324–330. https://dx.doi.org/10.11646/zootaxa.3731.3.2 25277574 10.11646/zootaxa.3731.3.2

[B13] Wieczorek K Mróz E (2006) The structure of the male reproductive system of selected Hemiptera groups. Acta Biologica Cracoviensia 48(suppl. 1): 72.

[B14] Wieczorek K Płachno BJ Świątek P (2011) Comparative morphology of the male genitalia of Aphididae (Insecta, Hemiptera): part 1. Zoomorphology 130(4): 289–303. https://dx.doi.org/10.1007%2Fs00435-011-0134-z 22131642 10.1007/s00435-011-0134-z PMC3213338

[B15] Wieczorek K Świątek P (2007) Clasification of the drepanosiphine aphids (Hemiptera, Aphidoidea: Phyllaphidinae, Calaphidinae) in the light of anatomical research. Genus International Journal of Invertebrate Taxonomy 14: 63–65.

[B16] Wieczorek K Świątek P (2008) Morphology and ultrastructure of the male reproductive system of the woolly beech aphid Phyllaphis fagi (Hemiptera: Aphididae: Phyllaphidinae). European Journal of Entomology 105: 707–712. https://dx.doi.org/10.14411/eje.2008.096

[B17] Wojciechowski W (1977) Procesy oligomeryzacji w budowie męskiego układu rozrodczego miodownic (Homoptera, Lachnidae). Prace Naukowe USl (Katowice) 3: 140–164.

[B18] Wojciechowski W (2003) A monograph of the Palearctic Pterocommatinae (Aphididae, Aphidinea, Hemiptera). Wydawnictwo Uniwersytetu Śląskiego, Katowice, 112 pp.

